# Impact of zinc and iron agronomic biofortification on grain mineral concentration of finger millet varieties as affected by location and slope

**DOI:** 10.3389/fnut.2023.1159833

**Published:** 2023-05-05

**Authors:** Demeke Teklu, Dawd Gashu, Edward J. M. Joy, R. Murray Lark, Elizabeth H. Bailey, Lolita Wilson, Tilahun Amede, Martin R. Broadley

**Affiliations:** ^1^Center for Food Science and Nutrition, Addis Ababa University, Addis Ababa, Ethiopia; ^2^Faculty of Epidemiology and Population Health, London School of Hygiene and Tropical Medicine, London, United Kingdom; ^3^Sustainable Soil and Crop, Rothamsted Research, Hertfordshire, United Kingdom; ^4^School of Bioscience, University of Nottingham, Sutton Bonington Campus, Leicestershire, United Kingdom; ^5^Alliance for a Green Revolution in Africa (AGRA), Sustainably Growing Africa’s Food Systems, Nairobi, Kenya

**Keywords:** agronomic biofortification, Iron, fertilizer, finger millet, micronutrient deficiencies, Zinc

## Abstract

**Background:**

Food crop micronutrient concentrations can be enhanced through agronomic biofortification, with the potential to reduce micronutrient deficiencies among rural population if they have access to fertilizers. Here we reported the impact of agronomic biofortification on finger millet grain zinc (Zn) and iron (Fe) concentration.

**Methods:**

A field experiment was conducted in farmers’ fields in Ethiopia in two locations; over two seasons in one district (2019 and 2020), and over a single season (2019) in a second district. The experimental design had 15 treatment combinations comprising 3 finger millet varieties and 5 soil-applied fertilizer treatments: (T1) 20 kg ha^−1^ FeSO_4_ + 25 kg ha^−1^ ZnSO_4_ + NPKS; (T2) 25 kg ha^−1^ ZnSO_4_ + NPKS; (T3) NPKS; (T4) 30% NPKS; (T5) 20 kg ha^−1^ FeSO_4_ + NPKS. The treatments were studied at two slope positions (foot and hill), replicated four times in a randomized complete block design.

**Results:**

Grain Zn concentration increased by 20% in response to Fe and Zn and by 18.9% due to Zn addition. Similarly, grain Fe concentration increased by 21.4% in T1 and 17.8% in T5 (Fe). Zinc fertilizer application (*p* < 0.001), finger millet variety (*p* < 0.001), and an interaction of Fe and Zn had significant effect on grain Zn concentration. Iron fertilizer (*p* < 0.001) and interactive effect of Fe fertilizer and finger millet variety (*p* < 0.01) had significant effects on grain Fe concentration. Location but not slope position was a source of variation for both grain Zn and Fe concentrations.

**Conclusion:**

Soil application of Zn and Fe could be a viable strategy to enhance grain Zn and Fe concentration to finger millet grain. If increased grain Zn and Fe is bioavailable, it could help to combat micronutrient deficiencies.

## Introduction

Micronutrient deficiencies (MNDs), which affect more than 2 billion people globally (1), is predominantly a result of intake of monotonous diets dominated by foods of low nutrient content (2). MNDs are more prevalent in developing countries where the diet is dominated by staple crop-based foods (3). In Ethiopia, a high prevalence of Zn (72%) and Fe (34.4%) deficiencies has been reported based on biomarkers of status (4). The nutritional quality including Zn and Fe content of cereals (5) also varies geospatially in Ethiopia. Zinc and Fe deficiencies can lead to impaired physical growth and cognitive functions, reduced resistance to infections, metabolic disorders, and increased prenatal morbidity (6).

MNDs can be addressed through different program that are either targeted on enhancing vitamin and mineral intake, or on reducing loss of nutrients from the body. Strategies can include dietary diversification, food fortification, and supplementation. In addition, public health measures such as deworming, vaccination, improved water, sanitation and hygienic practices, improved health care system and nutrition education are crucial interventions to prevent MNDs (7). Each strategy has strengths and limitations, and they should be considered in the context of local conditions. For example, dietary diversification is the most sustainable and preferred strategy since it can potentially address the root cause of MNDs. However, availability and affordability of the diversified foods can be barriers in resource poor societies. Food fortification can potentially have wider impact and be more cost-effective compared to supplementation. However, food fortification is limited to the centrally processed foods and thus difficult to address societies that are dependent on local food sources. Supplementation is preferred when the MND is severe and there is a need to provide the quickest improvements, although can present many logistical challenges (8).

Agronomic biofortification is a strategy to increase micronutrient content in the edible part of food crops through the application of mineral fertilizers (9). This approach can enrich food crops with multiple elements at a time and can reach resource poor rural populations, providing they have access to fertilizers. On the other hand, repeated and excess use of mineral fertilizers may cause soil and water contamination over time suggesting the need for regular monitoring of the environmental impact of agronomic biofortification (10). For agronomic biofortification to be effective, targeting food crops and varieties known to well adapt in their local environment is important (11).

Finger millet has several agronomic merits that can make it well-adapted to certain environments, including tolerance to moisture stress and soil acidity as well as resistance to disease (12). Finger millet also has appreciable nutritional content, e.g., high calcium (Ca) concentration 450 mg 100 g^−1^ (10-fold greater than milk on a volume basis) (5, 13). It is also a good source of other micronutrients (5) and protein (15.58%) (14). In addition, finger millet grain is gluten free, which makes it more attractive than wheat to some consumers (15). Finger millet is an indigenous crop to Ethiopia, and the sixth most important cereal crop after teff, wheat, maize, sorghum, and barley. It is produced on ~480,000 hectare of land, yielding about 1.2 M tonnes *per annum* (16). To our knowledge, there is no available previous data on finger millet for the effect of Zn, Fe, or combined fertilizers on grain Zn and Fe concentration. Therefore, this study aims to determine the potential impact of agronomic biofortification on finger millet grain Zn and Fe concentration. In addition, this study aims to investigate influence of varietal and environmental factors on Zn and Fe biofortification response. For example, it has been observed that the amount of plant available Zn can vary in different landscape positions of Ethiopian mixed cereal cropping systems (17).

## Materials and methods

### Field experiment

Agronomic biofortification experiments with Zn and Fe fertilizers were carried out in farmer fields, in two districts (locally known as *Woreda*) in the Amhara and Oromia regions, Ethiopia: Gojjam (11°41′54”N 37°29′79″E foot slope and 11°40′23”N 37°30′29″E hill slope) and Arsi Negelle (7°19′38”N 38°38′54″E foot slope and 7°18′43”N 38°39′57″E hill slope), respectively. According to the agro-ecological classification of Ethiopia, both sites are characterized as sub-humid midlands located between 1,500 and 2,300 m.a.s.l. and receive an average annual rainfall of 800–1,200 mm (18). The experiment consisted of 15 treatment combinations: 3 finger millet varieties (Diga-01, black grain colour; Urji white grain colour; Meba, brown grain colour) and 5 soil-applied fertilizer treatments as indicated below:

*T1*: 25 kg ha^−1^ ZnSO_4_7H_2_O, 20 kg ha^−1^ FeSO_4_7H_2_O, 131 kg ha^−1^ NPS, 60 kg ha^−1^ potassium (K), 54 kg ha^−1^ urea.

*T2*: 25 kg ha^−1^ ZnSO_4_7H_2_O, 131 kg ha^−1^ NPS, 60 kg ha^−1^ K, 54 kg ha^−1^ urea.

*T3*: 131 kg ha^−1^ NPS, 60 kg ha^−1^ K, 54 kg ha^−1^ urea (control).

*T4*: 30% rate of T3 (Negative control).

*T5*: 20 kg ha^−1^ FeSO_4_7H_2_O, 131 kg ha^−1^ NPS, 60 kg ha^−1^ K, 54 kg ha^−1^ urea.

Note: The negative control was included to observe the dilution effect. This is because reports show that considerable number of farmers reported to use very low amount of fertilizer as compared to the recommendation (19).

A randomized completed block design (RCBD) was used with 4 replications. The plot size was 4 m x 4 m, with gangways of 1 m width between plots while the distance between the block was 0.5 m. The experiment was repeated for two seasons but only at Arsi Negelle (due to Covid-19 pandemic travel restriction in Gojjam). Different farms were used in each year, sowed between mid-June and mid-July. Harvesting of the trial crop was on the first week of November and end November at Arsi Negelle and Gojjam, respectively.

### Sample collection

Soil samples for mineral analysis were collected from a 60 m^2^ circular plot in each of the experimental fields from both locations. Five sub-sample sites were located, the first at the centre, then two sub-sample points were selected at locations on a line through the plot centre along the crop rows, and two on a line orthogonal to the first through the plot centre. The ‘long’ axis of the sample array (with sample locations at 5.64 m and 4.89 m) was oriented in the direction of crop rows with the ‘short axis’ (with sample locations at 3.99 m and 2.82 m) perpendicular to the crop rows. A single soil sample was collected (to 20 cm depth) at each of the five sub-sample points with a Dutch auger with a flight of length 150 mm and diameter 50 mm. Any plant material adhering to the auger was carefully removed, and the sub-samples were aggregated and stored in a single bag. The detail procedure on soil sample collection, processing and mineral analysis follows Gashu et al. (20).

Matured and dried finger millet crop fingers were taken from each plot and the crop samples were hand threshed to produce approximately 1 kg of grain representing a sample and whole-grain samples were packed in sample envelope (5).

### Sample preparation

Whole-grain samples were air-dried in sample bags. The samples were ground in a domestic stainless-steel coffee grinder, which was wiped clean before use and after each sample with a non-abrasive cloth. All preparations were done away from sources of soil and dust contamination. A 20 g subsample (following a representative quartering system) of the ground finger millet was then shipped to the University of Nottingham, UK. Soil samples were oven-dried at 40°C for 24–48 h depending on the moisture content of the soil. Preparation took place in a soil laboratory to avoid cross-contamination. Plant material and stones were removed from soil samples, which was then disaggregated and sieved to pass through 2 mm (5). This material was representatively quartered to produce subsamples. A 150 g subsample of soil was poured into a self-seal bag, labeled and shipped to the UK for analysis in the laboratories at the University of Nottingham.

### Soil and grain mineral analysis

The soil samples were digested using Aqua-Regia for mineral analysis (20). A certified reference material (CRM Wageningen, WEPAL ISE-850, Calcareous Soil) was digested and analysed for mineral concentration for quality control purpose of the method. Operational blanks were also analysed at the same time following the same procedure to determine limits of detection (LODs). A three-step sequential extraction scheme for the fractionation of sulfur (S) was followed, using 0.01 M KNO_3_, 0.016 M KH_2_PO_4_, and 10% tetra methyl ammonium hydroxide (TMAH) to determine soluble, exchangeable, and organically bound S fractions, respectively. The detailed procedure for soil mineral analysis and three-step sequential extraction for S is reported elsewhere (20–22).

Hot plate acid digestion was employed for grain sample digestion using methods described in Kumssa et al. (22). Briefly, 0.2 g of finger millet grain samples were weighed in digestion tubes and placed into heating blocks (Multicube 48, Anton Paar Ltd., UK). Then, 8 mL of concentrated HNO_3_ (trace metal grade, Fisher Chemical, United States) was added to each tube and left for 30 min at room temperature. The samples were heated for 2 h at 115°C, left to cool for 10 min, and the samples were diluted to 50 mL using milliQ water (18.2 MΩ cm; Fisher Scientific). A 1 mL aliquot was transferred into an inductively coupled plasma tube and diluted again to 10 mL using milliQ water. A certified reference material (CRM Wheat 1567b, National Institute of Standards and Technology, Gaithersburg, MD, United States) was used to determine % recovery. Operational blanks were also analysed to determine limits of detection (LODs). Soil and crop sample mineral concentrations were analysed by Inductively Coupled Plasma Mass Spectrometry (ICP-MS, Thermo Scientific, Germany).

### Statistical analysis

Data were analysed using R software version 3.3.2. The data were presented as mean ± standard deviation (SD). The data were analysed with a linear mixed model to compare mineral concentrations among control and fortified grain samples. Slope, fertilizer, and variety were treated as fixed effects whereas season, block within farm, farm within location, and location were treated as random effects. For logistical reasons treatments and varieties were randomized within farms, and farms were selected from slope positions within locations. The replication for slope position is thus very limited, and the model is prone to singularities. For several variables, slope was therefore dropped as a fixed effect and treated as a random effect for grain Fe concentration data. Then, variance component for the slope random effect was checked to determine how important slope might be relative to other factors. Skewness of the residuals for grain Zn and Fe concentration data and histograms were examined to decide whether any transformation was required before proceeding to interpretation of the outputs (see Supplementary material).

The fixed effect for treatments can be partitioned into four orthogonal contrasts, selected prior to data analysis, to test specific hypotheses. These were as follows.

*C1*: The comparison between the mean grain Zn for the 0.3NPKS treatment and all the treatments with NPKS at recommended rate.

*C2*: Within the full NPKS rate, the Fe main effect (difference between treatments with Fe and no Fe).

*C3*: Within the full NPKS rate, the Zn main effect (difference between treatments with Zn and no Zn).

*C4*: The Fe/Zn interaction: does the response to Zn depend on the level of Fe and vice versa.

## Results

**Figure 1:**
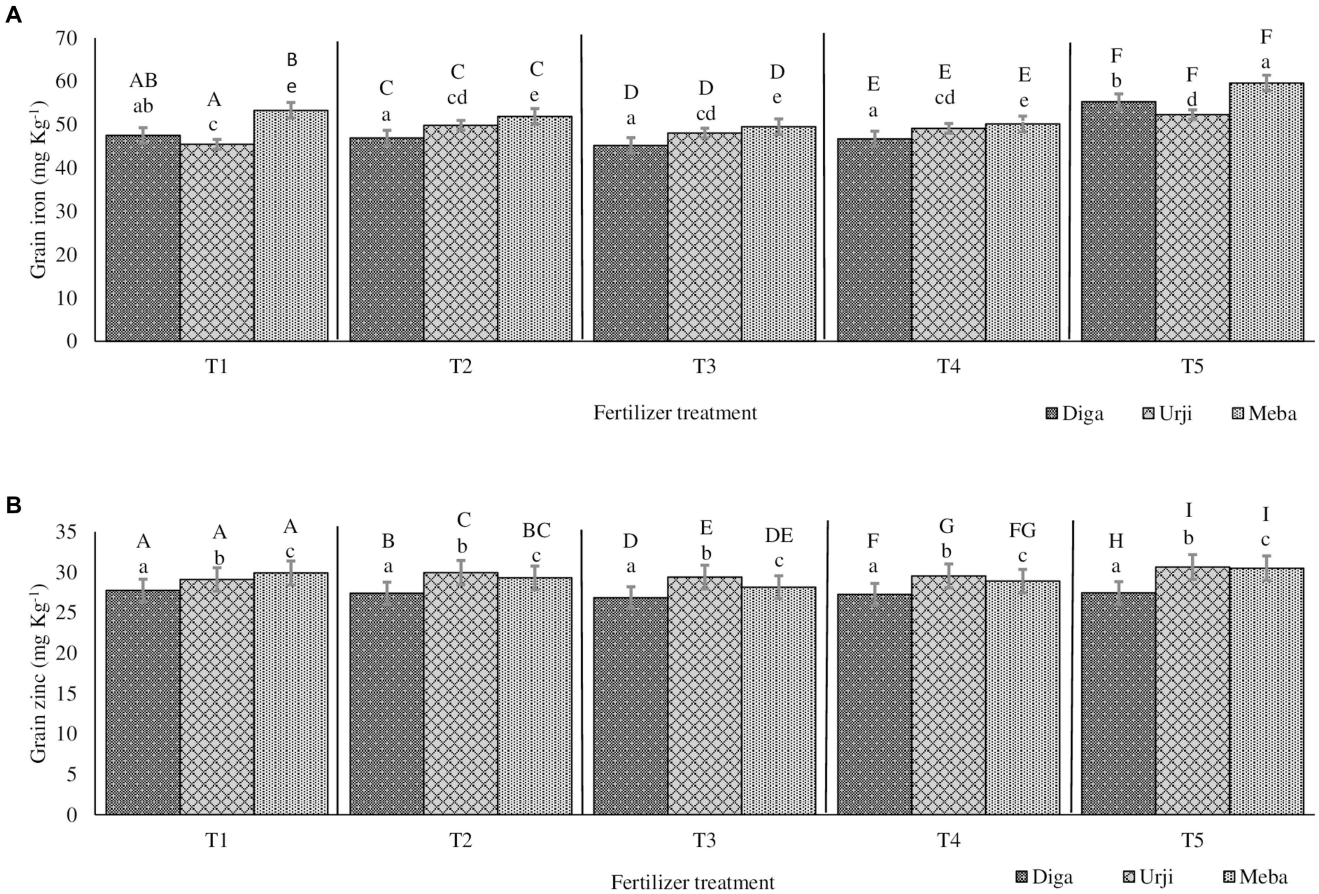
Grain iron **(A)** and zinc **(B)** concentration of finger millet at Arsi Negelle as affected by genotype and zinc and iron fertilization T1: 25 kg ZnSO_4_7H_2_O, 20 kg FeSO_4_7H_2_O, 131 kg NPS, 60 kg K, 54 kg urea ha^−1^; T2: 25 kg ZnSO_4_7H_2_O, 131 kg NPS, 60 kg K, 54 kg urea ha^−1^; T3: 131 kg NPS, 60 kg K, 54 kg urea ha^−1^; T4: 30% of T3; T5: 20 kg FeSO_4_7H_2_O, 131 kg NPS, 60 kg K, 54 kg urea ha^−1^. Columns designated by different lowercases (a, b, c, d, e) have significantly different response to fertilizer treatment for single genotype. Columns designated by different uppercases (A, B, C, D, E, F, G, H, I) have significantly different response to genotypes for single fertilizer treatment.

**Figure 2:**
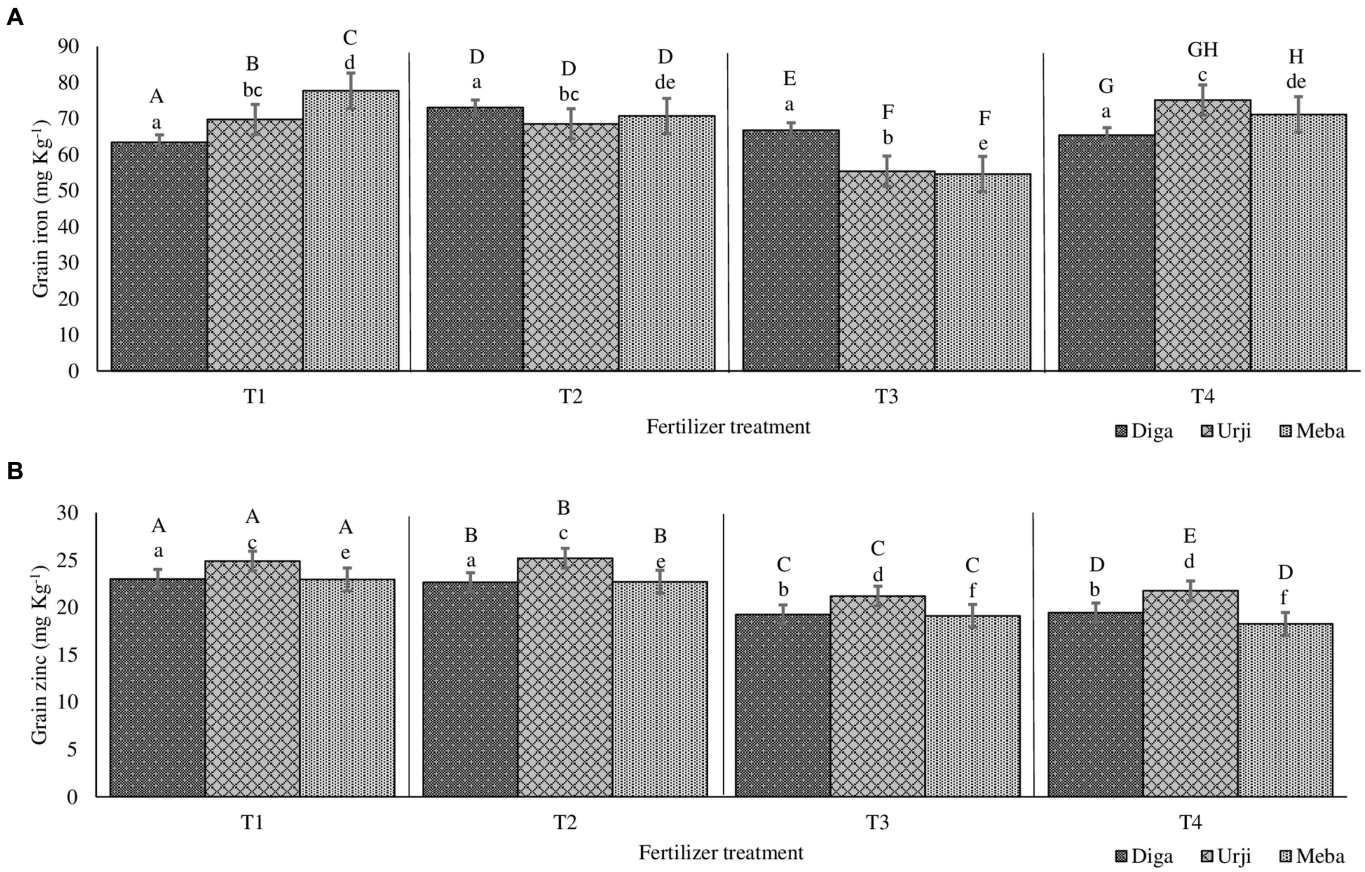
Grain iron **(A)** and zinc **(B)** concentration of finger millet at Gojjam as affected by genotype and zinc and iron fertilization T1: 25 kg ZnSO_4_7H_2_O, 20 kg FeSO_4_7H_2_O, 131 kg NPS, 60 kg K, 54 kg urea ha^−1^; T2: 25 kg ZnSO_4_7H_2_O, 131 kg NPS, 60 kg K, 54 kg urea ha^−1^; T3: 131 kg NPS, 60 kg K, 54 kg urea ha^−1^; T4: 30% of T3; T5: 20 kg FeSO_4_7H_2_O, 131 kg NPS, 60 kg K, 54 kg urea ha^−1^. Columns designated by different lowercases (a, b, c, d, e, f) have significantly different response to fertilizer treatment for single genotype. Columns designated by different uppercases (A, B, C, D, E, F, G, H, I) have significantly different response to genotypes for single fertilizer treatment.

Mineral concentrations of soil samples from both trial locations at each slope position is presented in Table 1. Calcium, potassium, boron, sulfur, and iron content of soil samples were significantly different among the two locations and slope positions. The recovery for all minerals is between the acceptable ranges (85–120%). Fe and Zn agronomic biofortification response of finger millet samples for each fertilizer treatment at different location and slope position is indicated in Figures 1, 2. The recovery percentage for grain Fe and Zn concentration was 91.0 and 96.4%, respectively.

**Table 1:** Mineral concentrations (mg/kg) of soil from the finger millet agronomic biofortification experimental sites in Ethiopia.

Location	Slope	B	Mg	P	S	K	Ca	Fe	Zn
Arsi-Negelle	Foot	3.9 ± 0.79^A^	2052 ± 47	2061 ± 49	122.7 ± 0.4	3,227 ± 185^A^	4,662 ± 481^A^	26,918 ± 1149^A^	89 ± 7
	Hill	3.0 ± 0.70^B^	1728 ± 240	1725 ± 240	105.8 ± 7.3	2,729 ± 448^B^	4,050 ± 918^B^	23,952 ± 1804^B^	105 ± 10
	Mean	3.4 ± 0.95^a^	1890 ± 259^a^	1893 ± 264^a^	114.3 ± 10.3^a^	2,978 ± 464^a^	4,356 ± 870 ^a^	25,435 ± 2320^a^	97 ± 13^a^
Gojjam	Foot	1.0 ± 0.37^C^	1731 ± 131	1731 ± 131	136.6 ± 6^C^	934 ± 33^C^	1,185 ± 149^C^	107,973 ± 3372^C^	81 ± 4
	Hill	0.1 ± 0.08^D^	1,597 ± 167	1,597 ± 167	207.1 ± 42.8^D^	859 ± 85^D^	1,668 ± 430^D^	124,304 ± 5913^D^	104 ± 11
	Mean	0.55 ± 0.5^b^	1,664 ± 180^a^	1,664 ± 180^a^	171.8 ± 47.3^b^	897 ± 82^b^	1,426 ± 440^b^	116,138 ± 10383^b^	92 ± 16^a^

Results labeled with different small letters are significantly different for location and those with different capital letter are significantly different for slope at the 0.05 probability level.

Grain Zn concentration was significantly different (*p* < 0.001) between fertilizer treatments (see Supplementary materials). There was 20 and 18.9% increase in grain Zn concentration in response to application of combined Fe and Zn, and Zn-only fertilizers, respectively (Figures 1, 2). There was no significant difference in grain Zn concentration between 30% NPKS and NPKS at recommended rate. Irrespective of fertilizer treatment, location and slope position, finger millet varieties differed significantly (*p* < 0.001) with respect to Zn concentration, showing the average result of Diga-01 < Meba < Urji (Figure 2). There is no evidence for an interaction between fertilizer and variety effect on grain Zn concentration (see Supplementary materials).

The orthogonal contrasts provide strong evidence (*p* < 0.001) for main effect of Zn fertilizer on grain Zn concentration as well as variety effect, some evidence for an interaction of Fe and Zn (*p* < 0.05), moderate evidence (*p* < 0.01) for a difference between the 30% NPKS and NPKS at recommended rate treatments (see Supplementary materials). Therefore, the application of Zn fertilizer significantly improves the grain Zn concentration while it is slightly dependant on the level of Fe fertilizer. Location was a source of variation for grain Zn concentration with Arsi Negelle having larger grain Zn concentration than Gojjam (see Supplementary materials). Despite soil Zn concentrations were significantly different among slope positions, slope (having smaller variance [0.3155] than block, location and season) was not the major source of variation of grain Zn concentration (see Supplementary materials). Therefore, block within the farm, slope position and season shows a negligible effect on grain Zn concentration.

Grain Fe concentration was significantly different (*p* < 0.001) between fertilizer treatments. NPKS shows the lowest (53.25 mg kg^−1^) and Zn-only fertilizer having the highest (60.2 mg kg^−1^) grain Fe concentration (Figures 1, 2). There was an average 21.4 and 17.8% enhancement of grain Fe concentration due to application of FeSO_4_7H_2_O and ZnSO_4_7H_2_O (21.4%) and FeSO_4_7H_2_O alone (17.8%) fertilization, respectively (Figures 1, 2). There was no significant difference in grain Fe concentration between 30% NPKS and NPKS application at recommended rate. As for grain Zn concentration, irrespective of fertilizer treatment, location and slope position, grain Fe concentration significantly (*p* < 0.01) differed between the varieties ranging between 53.6 and 57.2 mg kg^−1^ (Figures 1, 2). However, there was no evidence for an effect of slope position and an interaction of fertilizer and variety effect (see Supplementary materials). Thus, the fertilizer main effect was replaced by four orthogonal contrasts (slope and interaction effect dropped).

The orthogonal contrasts provide strong evidence (*p* < 0.001) for interaction of Fe and Zn effect on grain Fe concentration, suggesting that the response to Fe strongly depends on the level of Zn fertilizer application (see Supplementary materials). The Analysis of Variance indicates moderate evidence (*p* < 0.01) for an effect of Fe fertilizer application as well as variety on grain Fe concentration (see Supplementary materials). Location was a source of variation in grain Fe concentration, with Gojjam having larger grain Zn concentration than Arsi Negelle (see Supplementary materials). However, block within the farm as well as farm within the location shows negligible effect on grain Fe concentration (see Supplementary materials).

## Discussion

The present study investigated the impact of Zn and Fe agronomic biofortification on finger millet grain Zn and Fe concentrations. Irrespective of variety, location and slope positions, finger millet grain Zn and Fe concentration increased by 18.9–20% and 17.8–21.4%, respectively, as a result of Zn and Fe agronomic biofortification. This suggest that agronomic biofortification of finger millet can be an effective supplementary strategy to reduce Fe and Zn deficiency, if the increased Fe and Zn in grains are bioavailable. Our finding also indicates that finger millet’s response to Zn and Fe agronomic biofortification significantly was affected by variety and location, but not slope positions.

### Zinc fertilizer increased grain Zn concentration in finger millet

The current experiment shows strong evidence that Zn fertilization effectively enhances grain Zn concentration of finger millet. Joy et al. (23) reported an incremental effect of soil application of Zn fertilizer on Zn concentrations of maize (20%), rice (7%) and wheat (19%) in 10 African countries. In addition, an experiment by Botoman et al. (24) and Manzeke et al. (25) showed that maize grain Zn concentration was increased by 15 and 44.5% due to the application of 30 kg and 45 kg ZnSO_4_ ha^−1^, respectively. Similarly, 29% maize grain Zn enhancement was observed as a result of 50 kg ZnSO_4_7H_2_O ha^−1^ fertilizer application (26). Another research showed 17.7% increase in wheat Zn concentration in response to soil application of up to 37.5 kg ZnSO_4_ ha^−1^ (27). Pal et al. (28) also reported 21.3% increase of Zn in chickpea through 25 kg ZnSO_4_ ha^−1^fertilizer application. Another study from India also showed an increase of 26.5% rice Zn concentration by applying 5 kg ZnSO_4_ fertilizer per hectare (29). Similar increase in Zn concentration of wheat grain following soil Zn application was also seen in Australia (30), Turkey (31) and India (32). Contrary to the finds of previous studies reporting positive effects of nitrogen on grain Zn concentration (25, 26), in the present study nitrogen had no significant effect on grain Zn concentration.

Variations in relative response of grain Zn concentration to Zn agronomic biofortification was observed between and among the present and previous studies could be attributed to several factors including due to differences in crops’ ability to relocalize and remobilize Zn into the grain (33, 34). Wu et al. (35) also reported that rice Zn concertation density in rice grain was closely associated with the ability to translocate Zn from old tissues to new tissues at both early and late growth stages and with phloem remobilization of Zn from leaves and stems to grains. Phloem mobility of each element greatly affects the amount of element remobilization in plants (36), and Zn showed good remobilization *via* phloem mobility (35). In addition, grain Zn concentration also significantly decreased as a result of elevated CO_2_ (37, 38) but increased as a result of heat stress (39), however the mechanism is still unknown. Another study reported that phosphorus can negatively affect Zn absorption by inhibiting colonization hence, impaires mycorrhizal uptake pathway (40, 41). Nitrogen significantly enhances Zn absorption in plants (42, 43), probably, by balancing charge which contribute to higher accumulation of cationic nutrients like Zn (42). Nitrogen also increases the activity of transporter proteins and nitrogenous compounds that helps to maintain Zn root uptake and shoot translocation (44, 45).

### Iron fertilizer increased grain Fe concentration in finger millet

The current experiment shows strong evidence that Fe fertilization effectively enhances grain Fe concentration of finger millet. There is no available data on agronomic biofortification of Fe on finger millet results, however, on other crops available research showed a significant increase on wheat due to the application of Fe fertilizer. Results of a greenhouse experiment reported a 19.4% increase in grain Fe concentration due to soil application of 10 mg of FeSO_4_ kg^−1^ of soil (42) or 43% increase in response to soil application of 75 FeSO_4_ kg ha^−1^ (27).

Pahlavan-Rad and Pessarakli (46) observed a 36% grain Fe concentration increase due to 1% of FeSO_4_ foliar application at stem elongation and flowering stages. Manzeke-Kangara et al. (47) also reported about 83% increase in finger millet grain Fe concentration due to foliar Fe-EDTA application at vegetative growth and flowering stage.

Agronomic biofortification of Fe is less well studied compared to Zn. Fe biofortification only moderately increase grain Fe concentration as compared to Zn biofortification. This might be associated to the fact that Fe has poor phloem mobility (48, 49). Also, when applied to calcareous soils, Fe is rapidly converted into unavailable forms (48, 49).

### Interactions between Zn and Fe fertilizers on finger millet grain Zn and Fe concentration

Enhancement of 13 and 5.5% grain Fe and Zn concentration, respectively, were observed due to Fe and Zn interaction effect. Similarly, Pahlavan-Rad and Pessarakli (46) also reported an 8 and 13% increase of wheat grain Fe and Zn concentration, respectively due to Fe and Zn interaction. Zinc fertilizers also resulted in Fe accumulation in soybean roots and increased root to fruit Fe translocation in tomato plants (50). These might be due to both Zn and Fe deficiency in plant is signaled by the same gene (51, 52). Also, Fe-and Zn-regulated transporter encoding genes expression in roots and shoots is induced at the transcriptional level by Zn and/or Fe availability (53–55), which suggests these genes may control the uptake and homeostasis of Fe and Zn (56). However, additional experiments are required to understand the mechanism of Zn and Fe interactions.

### Effect of finger millet genotype and response to Zn and Fe fertilizer on grain Zn and Fe concentration

Response to agronomic biofortification of Zn and Fe in current experiment was significantly influenced by finger millet variety. In the present study, Diga-01 variety accumulated the lowest Zn concentration while Meba variety had the highest Zn. Though, the genetic variability in mineral concentration of finger millet has been previously reported (57), there is no available data on the finger millet’s genetic response to Zn and Fe fertilization. However, Wissuwa et al. (58) from Philippines showed that rice genotypes differ greatly in their response to foliar Zn treatments. Similarly, significant varietal response to the soil application of Zn fertilizer on rice grain Zn concentration was reported (59). Despite a similar root uptake rate or shoot accumulation of minerals, genotypic differences influence grain mineral concentrations (60).

Stability of a trait over different locations is an important factor in breeding programs (61). When a highly promising genotype for grain Zn and Fe concentration is identified, special attention should be paid to how the grain Zn and Fe concentration of that genotype vary with soil type (62). In the current experiment Meba and Urji finger millet varieties exhibited stability over the two locations on grain Fe and Zn concentration, respectively.

### Effect of location on grain Zn and Fe concentration in finger millet

Location had significant effect on grain Zn and Fe concentration of finger millet. Similarly, their subnational scale data from Ethiopia and Malawi, Gashu et al. (5) reported location as a source of variability in cereal grain mineral concentration.

## Conclusion

The application of 25 kg ZnSO_4_7H_2_O and 20 kg FeSO_4_7H_2_O per hectare along with recommended rate of NPKS enhances grain Zn and Fe concentration of finger millet and can offer an effective option to increasing Zn and Fe concentration among consumers. However, social acceptability of agronomically biofortified food and technological and economic feasibility of application of mineral blended fertilizers and social acceptability of agronomically biofortified foods in resource poor settings could be a challenge and warrants further studies are important. In addition, crop grains such as finger millet have high amount of fiber and antinutritional factors that reduce bioavailability of minerals hence, evaluation of the bio-accessibility and bioavailability of biofortified crops is essential. Furthermore, use of mineral fertilizers could be contamination risks to the environment from the minerals of interest and contaminants in the mineral mixtures. For example, Stuart et al. (10) reported extra addition of 28 tonnes of Cu to the soil each year from use of Cu fertilizer for cereal biofortification in parts of the United Kingdom.

## Data availability statement

The raw data supporting the conclusions of this article will be made available by the authors, without undue reservation.

## Author contributions

DT, DG, EJ, RL, TA, and MB: conceptualization. DT: data collection, supervision, and Original draft preparation. DT and EB: sample preparation and analysis. DT, DG, and RL. Statistical analysis. DT, DG, EJ, MRL, EB, LW, TA, and MB: writing—review and editing. All authors contributed to the article and approved the submitted version.

## Funding

This work was also supported, in part, by the Bill & Melinda Gates Foundation [INV-009129]. Under the grant conditions of the Foundation, a Creative Commons Attribution 4.0 Generic License has already been assigned to the Author Accepted Manuscript version that might arise from this submission. Funding was also provided by USAID: Feed the future. The funders had no role in the design, execution, analyses or interpretation of the data.

## Conflict of interest

The authors declare that the research was conducted in the absence of any commercial or financial relationships that could be construed as a potential conflict of interest.

## Publisher’s note

All claims expressed in this article are solely those of the authors and do not necessarily represent those of their affiliated organizations, or those of the publisher, the editors and the reviewers. Any product that may be evaluated in this article, or claim that may be made by its manufacturer, is not guaranteed or endorsed by the publisher.
