# Supplementary material for: Impact of zinc and iron agronomic biofortification on grain mineral concentration of finger millet varieties as affected by location and slope
Source: Front Nutr. 2023 May 5;10:1159833. doi: 10.3389/fnut.2023.1159833 (PMC10195999; doi:10.3389/fnut.2023.1159833)
Supplement: Supplementary file 1 [file Data_Sheet_1.docx]

In principle the full model for these data would be:

Fixed effects, slope, fertilizer, variety

Random effects, Year and block within farm within location.

However, the way the randomization was done in the end, with all slope positions in a single farm within any location, it can be difficult to estimate the full model because of singularities. These will not automatically arise but will for some variables. Then, the strategy has been to run the full model where it can be, but to drop slope as a fixed effect where problems arise. Then one can examine the variance component for the farm random effect to get an idea of how important slope might be relative to other factors, as it will be a component of the between farm variance component in models where it is not a fixed effect.

**Zinc**

Exploratory analysis of the raw data. Summary statistics are output in R


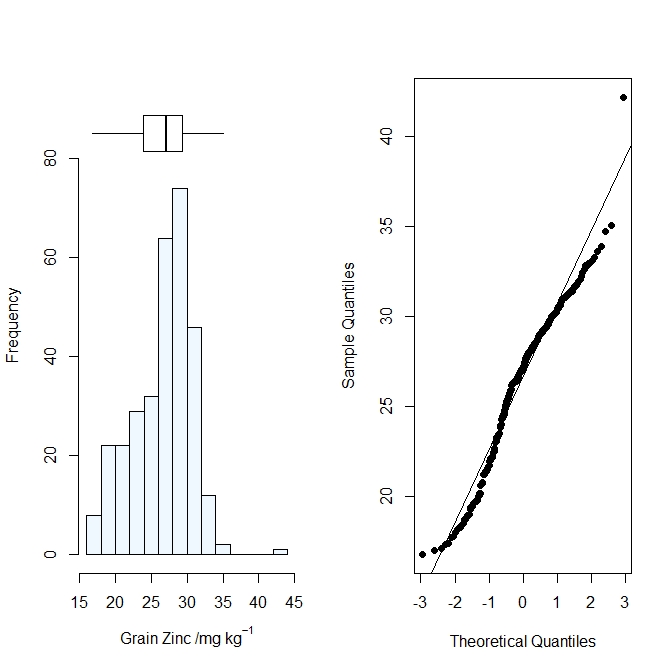


Analysis of variance for first basic model

> anova(model,refit=F)

Type III Analysis of Variance Table with Satterthwaite's method

Sum Sq Mean Sq NumDF DenDF F value Pr(>F)

FERTILIZER 167.939 41.985 4 274.68 11.3002 1.672e-08 ***

VARIETY 243.631 121.815 2 274.40 32.7865 1.707e-13 ***

FERTILIZER:VARIETY 27.619 3.452 8 275.85 0.9292 0.4926

Note there is evidence for differences among the fertilizer treatments, and between the varieties, but no evidence for an interaction. For this reason, further outputs are based on a model with this interaction dropped, specifically the following plots for fertilizer and variety effects


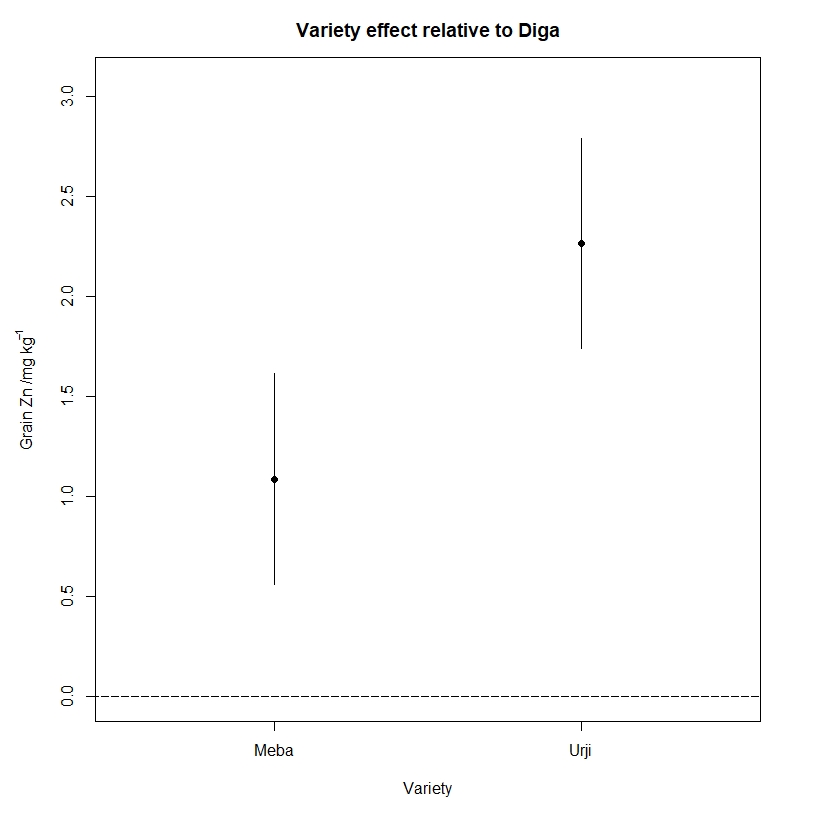


Finally, we run the model (interaction dropped) with the fertilizer main effect replaced by four orthogonal contrasts. These contrasts are as follows.

C1: The comparison between the mean grain Zn for the 0.3NPKS treatment and all the treatments with NPKS at recommended rate.

C2: Within the full NPKS rate, the Fe main effect (difference between treatments with Fe and no Fe)

C3: Within the full NPKS rate, the Zn main effect (difference between treatments with Zn and no Zn)

C4: The Fe/Zn interaction: does the response to Zn depend on the level of Fe?

> anova(model.r2)

Type III Analysis of Variance Table with Satterthwaite's method

Sum Sq Mean Sq NumDF DenDF F value Pr(>F)

C1 35.635 35.635 1 282.63 9.6062 0.0021348 **

C2 11.903 11.903 1 282.93 3.2088 0.0743113 .

C3 49.416 49.416 1 282.97 13.3211 0.0003125 ***

C4 18.363 18.363 1 282.98 4.9502 0.0268756 *

VARIETY 265.974 132.987 2 282.49 35.8493 1.334e-14 ***

---

Signif. codes: 0 ‘***’ 0.001 ‘**’ 0.01 ‘*’ 0.05 ‘.’ 0.1 ‘ ’ 1

So, we can see strong evidence for a main effect of Zn fertilizer, some evidence for an interaction of Fe and Zn, moderate evidence for a difference between the 30% NPKS and NPKS treatments and a strong variety effect. This interprets the two key plots above.

The summary function applied to this final model allows to examine the variance components for each random effect.

Note that the between farm variance is small (0.3155), smaller than season, location or block effects. This suggests that slope position is not a major source of variation in grain Zn content.

Random effects:

Groups Name Variance Std.Dev.

BLOCK_ID:(FARM_ID:LOCATION) (Intercept) 0.3290 0.5736

FARM_ID:LOCATION (Intercept) 0.3155 0.5617

LOCATION (Intercept) 19.4043 4.4050

YEAR (Intercept) 0.8907 0.9438

Residual 3.7096 1.9260

Supplementary Table 1. Effect of fertilizer type and finger millet variety on biofortified finger millet Zn concentration

| Factors | Sum Sq | Mean Sq | NumDF | DenDF | F value | Pr (>F) |
| --- | --- | --- | --- | --- | --- | --- |
| Fertilizer | 167.939 | 41.985 | 4 | 274.68 | 11.3002 | 1.672e^-08^ *** |
| Variety | 243.631 | 121.815 | 2 | 274.40 | 32.7865 | 1.707e^-13^ *** |
| Fertilizer: variety | 27.619 | 3.452 | 8 | 275.85 | 0.9292 | 0.4926 |

Significance codes: *** < 0.001

Supplementary Table 2. Type III Analysis of Variance with Satterthwaite's method for grain Zn concentration

|  | Sum Sq | Mean Sq | NumDF | DenDF | F value | Pr(>F) |
| --- | --- | --- | --- | --- | --- | --- |
| C1 | 35.635 | 35.635 | 1 | 282.63 | 9.6062 | 0.0021348** |
| C2 | 11.903 | 11.903 | 1 | 282.93 | 3.2088 | 0.0743113 |
| C3 | 49.416 | 49.416 | 1 | 282.97 | 13.3211 | 0.0003125*** |
| C4 | 18.363 | 18.363 | 1 | 282.98 | 4.9502 | 0.0268756* |
| Variety | 265.974 | 132.987 | 2 | 282.49 | 35.8493 | 1.334e^-14^*** |

Significance codes: *** <0.001; **< 0.01; *< 0.05

C1: The comparison between the mean grain Zn for the T4 (30% NPKS) and all the treatments (NPKS at recommended rate)

C2: Within the NPKS at recommended rate, the FeSO_4_7H_2_O main effect (difference between treatments with FeSO_4_7H_2_O and no FeSO_4_7H_2_O)

C3: Within the NPKS at recommended rate, the ZnSO_4_7H_2_O main effect (difference between treatments with ZnSO_4_7H_2_O and no ZnSO_4_7H_2_O)

C4: The FeSO_4_7H_2_O/ZnSO_4_7H_2_O interaction: does the response to FeSO_4_7H_2_O depend on the level of ZnSO_4_7H_2_O

Supplementary Table 3. The variance components of random effects for grain Zn concentration

| Groups | Name | Variance |
| --- | --- | --- |
| Block within the farm | Intercept | 0.3290 |
| Slope position | Intercept | 0.3155 |
| Location | Intercept | 19.4043 |
| Season | Intercept | 0.8907 |
| Residual |  | 3.7096 |

Supplementary Table 4. Effect of fertilizer type and finger millet variety on biofortified finger millet grain iron concentration, Ethiopia

|  | Sum Sq | Mean Sq | NumDF | DenDF | F value | Pr (>F) |
| --- | --- | --- | --- | --- | --- | --- |
| Slope position | 140.01 | 140.01 | 1 | 2.985 | 1.9644 | 0.256013 |
| Fertilizer | 1781.52 | 445.38 | 4 | 274.420 | 6.2488 | 7.959e-05*** |
| Variety | 719.14 | 359.57 | 2 | 273.683 | 5.0448 | 0.007055 ** |
| Fertilizer:variety | 1021.69 | 127.71 | 8 | 274.800 | 1.7918 | 0.078574. |

Significance codes: *** <0.001; **< 0.01; *< 0.05

Supplementary Table 5. Type III Analysis of Variance with Satterthwaite's method for grain Fe concentration

|  | Sum Sq | Mean Sq | NumDF | DenDF | F value | Pr(>F) |
| --- | --- | --- | --- | --- | --- | --- |
| C1 | 0.71 | 0.71 | 1 | 282.15 | 0.0097 | 0.9215000 |
| C2 | 679.75 | 679.75 | 1 | 283.29 | 9.3327 | 0.0024654** |
| C3 | 17.22 | 17.22 | 1 | 283.29 | 0.2364 | 0.6272052 |
| C4 | 987.87 | 987.87 | 1 | 283.13 | 13.5630 | 0.0002763*** |
| Variety | 738.34 | 369.17 | 2 | 281.78 | 5.0685 | 0.0068774** |

Significance codes: ***< 0.001; **< 0.01; *<0.05

C1: The comparison between the mean grain Fe for the T4 (30% NPKS) and all the treatments (NPKS at recommended rate),

C2: Within the NPKS at recommended rate, the FeSO_4_7H_2_O main effect (difference between treatments with FeSO_4_7H_2_O and no FeSO_4_7H_2_O),

C3: Within the NPKS at recommended rate, the ZnSO_4_7H_2_O main effect (difference between treatments with ZnSO_4_7H_2_O and no ZnSO_4_7H_2_O), and

C4: The FeSO_4_7H_2_O/ZnSO_4_7H_2_O interaction: does the response to FeSO_4_7H_2_O depend on the level of ZnSO_4_7H_2_O

Supplementary Table 6. The variance components of random effects for grain Fe concentration

| Groups | Name | Variance |
| --- | --- | --- |
| Block within the farm | Intercept | 12.16 |
| Farm within the location | Intercept | 21.51 |
| Location | Intercept | 172.56 |
| Residual |  | 72.84 |
